# Which difficulties do GPs experience in consultations with patients with unexplained symptoms: a qualitative study

**DOI:** 10.1186/s12875-019-1049-x

**Published:** 2019-12-29

**Authors:** Juul Houwen, Peter L. B. J. Lucassen, Anna Verwiel, Hugo W. Stappers, Willem J. J. Assendelft, Tim C. olde Hartman, Sandra van Dulmen

**Affiliations:** 1 Department of Primary and Community Care, Radboud University Nijmegen Medical Centre, Geert Grooteplein 21, 6525 EZ Nijmegen, The Netherlands; 2 Department of Primary and Community care, Donders Institute for Brain, Cognition and Behaviour, Radboud university medical center, Nijmegen, The Netherlands; 3 Faculty of Health and Social Sciences, University of South-Eastern Norway, Drammen, Norway; 4 NIVEL (Netherlands institute for health services research), Utrecht, The Netherlands

**Keywords:** Medically unexplained symptoms, Consultation, General practice, Communication

## Abstract

**Background:**

Many general practitioners (GPs) struggle with the communication with patients with medically unexplained symptoms (MUS). This study aims to identify GPs’ difficulties in communication during MUS consultations.

**Methods:**

We video-recorded consultations and asked GPs immediately after the consultation whether MUS were presented. GPs and patients were then asked to reflect separately on the consultation in a semi-structured interview while watching the consultation. We selected the comments where GPs experienced difficulties or indicated they should have done something else and analysed these qualitatively according to the principles of constant comparative analysis. Next, we selected those video-recorded transcripts in which the patient also experienced difficulties; we analysed these to identify problems in the physician-patient communication.

**Results:**

Twenty GPs participated, of whom two did not identify any MUS consultations. Eighteen GPs commented on 39 MUS consultations. In 11 consultations, GPs did not experience any difficulties. In the remaining 28 consultations, GPs provided 84 comments on 60 fragments where they experienced difficulties. We identified three issues for improvement in the GPs’ communication: psychosocial exploration, structure of the consultation (more attention to summaries, shared agenda setting) and person-centredness (more attention to the reason for the appointment, the patient’s story, the quality of the contact and sharing decisions). Analysis of the patients’ views on the fragments where the GP experienced difficulties showed that in the majority of these fragments (*n* = 42) the patients’ comments were positive. The video-recorded transcripts (*n* = 9) where the patient experienced problems too were characterised by the absence of a dialogue (the GP being engaged in exploring his/her own concepts, asking closed questions and interrupting the patient).

**Conclusion:**

GPs were aware of the importance of good communication. According to them, they could improve their communication further by paying more attention to psychosocial exploration, the structure of the consultation and communicating in a more person-centred way. The transcripts where the patient experienced problems too, were characterised by an absence of dialogue (focussing on his/her own concept, asking closed questions and frequently interrupting the patient).

## Background

Medically unexplained symptoms (MUS) are common in general practice. MUS are physical symptoms for which no pathological cause can be found after a proper examination. No underlying disease can be found for 3 to 11% of the presented symptoms [1–3]. MUS include a wide variety of unspecified symptoms, such as abdominal discomfort, dizziness, headaches and fatigue. Patients with severe MUS suffer from their symptoms, are functionally impaired, and are at risk of potentially harmful additional testing and treatment procedures [4].

General practitioners (GPs) have a central role in managing patients with MUS. GPs consider themselves as adequately positioned to manage these patients [5]. This is in line with MUS patients’ views, as they feel the need for continuity of care, one of the core values of primary care [6]. However, many GPs find MUS consultations challenging and experience problems in these consultations [7–10].

A meta-ethnography of 13 studies based on focus groups and individual interviews with GPs found that GPs struggle with the incongruence between patients’ symptom presentations and the explanatory models for biomedical disease [11]. It mentioned GPs’ inability to manage the problems. Finally, it described the congruent feelings of patients and GPs, in which both the GP and the patient have negative experiences, which may cause problems in the relationship. The review also showed that GPs frequently emphasised the importance of a good relationship [11]. Other MUS consultation studies showed that many GPs struggle to give a tangible explanation and feel pressured into applying somatic intervention [12–14]. Further, many GPs ignore psychosocial cues [15] and communicate less patient-centred than in consultations with medically explained symptoms, as they often do not explore in depth the patient’s reason for the encounter, their ideas and expectations about the symptoms [16].

The problems described in the meta-ethnography [11] have only been studied indirectly through semi-structured interviews or focus groups and are therefore subject to recall bias and social desirability bias. Moreover, interviews may not reflect actual behaviour during the consultation. To obtain more realistic information about this topic, there is a need for a study using observation of GPs during the consultations. Video-stimulated recall is a method that uses reflection on recorded data: video-recorded consultations are played back to the GP to stimulate their recall of their thoughts, feelings and attitudes and to discuss these elements [17]. The power of this method lies in its concrete and situational approach. To obtain more information about the problems that GPs experience in the communication in MUS consultations, we analysed their comments while they were watching their own videorecorded MUS consultation. These insights may help GPs to improve their communication skills. Therefore, the first aim of this study is to identify GPs’ difficulties in communication during MUS consultations.

However, it would be premature to use these findings as a basis for improvements in GPs’ communication in MUS consultations. Having the ideal model of the consultation in their mind and seeing complex patients on a tight timescale, most GPs viewing their own consultation will find something that they think could improve their communication. Furthermore, the GPs’ views about what they could do better may not match the perceptions and needs of MUS patients. Analysing patients’ views on the fragments of the consultation in which GPs experienced difficulties in the communication might give information about the relevance of these difficulties for patients. Therefore, the second aim of this study is to gain more insight into problems in the physician-patient communication when both the GP and the patient experience difficulties in this communication.

## Methods

We performed individual, video-stimulated recall sessions with GPs and MUS patients in which we asked them to - independently and individually – reflect on their own video-recorded consultation [17]. In earlier research, the video-recorded MUS patients had reflected on their own consultation while watching the video [6, 18]. Here, we describe the GPs’ experiences. We used the COREQ guideline for the reporting of this study [19]. The study was approved by the local research ethics committee. Written informed consent was obtained from all GPs and patients.

### Study sample

Video-recorded consultations and verbatim transcripts were collected as described previously [6]. Further, we asked the patient before the consultation to complete a questionnaire (patients’ expectations from their GP regarding communication); after the consultation, the same questionnaire had to be filled out (patients’ experiences from their GP regarding communication). Patients’ expectations and their experiences were measured using the QUOTE-COMM (Quality Of care Through the patients’ Eyes). Based on the data from questionnaires, we studied what patients expected from their GP regarding communication, what they experienced during the consultation and the extent to which GPs met patients’ expectation. We have described these results in another paper [20]. Here, we focused on the GPs’ difficulties in communication during MUS consultations.

### Procedure

GPs were invited to view the recordings of their MUS consultations and comment on these consultations (i.e. stimulated recall). We tried to do this shortly after the consultation, but were dependent on the GPs’ schedules. Whenever the GP indicated that he/she had experienced difficulties regarding the consultation, the interviewer went into this subject in more detail. After the detailed discussion, the next question was: “What could you have done differently?” This question was asked once more after showing the whole video (The interview guide is added as Additional file 1). We also asked the GP to point out the moment when they decided on the diagnosis of MUS and we interviewed them about the triggers for labelling the symptoms as MUS in this specific consultation. However, we described these findings in another study [21].

### Analysis

The audio-recorded interviews (GPs’ reflections on the video-recorded consultation) were transcribed verbatim in Atlas.ti, a software program for analysing qualitative data. Two researchers (JH, a trainee GP and PhD student, and AV, a medical student) independently selected the comments where GPs experienced difficulties or indicated they should have done something else. Disagreements about the selection were resolved by discussion. Three researchers (JH, AV, student and ToH, GP) read all selected comments several times to familiarize themselves with the data. They coded the text and identified categories independently. The categories were discussed in consensus meetings. During the analysis we constantly matched the developing categories with the transcripts according to the principle of constant comparison [22]. Analysis was inductive to ensure that the process was grounded in the data rather than in preconceptions. In the final stage of the analytical process we found no new codes or categories.

The same procedure was used with the patients. Patients in these MUS consultations were asked to reflect on the consultation in a semi-structured interview while watching a recording of their own consultation. In previous research, we had already analysed the relevant and important communication elements according to patients [6] and explored the problems patients experienced in communication [18]. From the video-recorded transcripts, we selected the fragments identified by both GP and patient as being suboptimal in terms of communication. Two researchers (JH and PL, a GP) read these transcripts and watched the corresponding video-recorded consultations several times to familiarise themselves with the data. They independently coded the difficulties in the communication and discussed these in a consensus meeting.

## Results

In previous research, we described the GPs characteristics and MUS patients more in detail [6]. Of the 16 GPs who did not participate, four were women, six practices were located in rural areas and the other 10 were located in the city. Of the 116 patients who did not want to participate, 42 were male and the mean age was 49 years.

### Suboptimal communication as perceived by GPs

In 11 consultations, the GP did not experience any difficulties. In the remaining 28 consultations, 16 GPs provided 84 comments about difficulties in the consultation. We identified three main themes: [1] psychosocial exploration, [2] the structure of the consultation, [3] person-centredness (Table 1).

**Table 1:** Overview of the different themes and quotes by GPs that relate to the different themes

GP reported difficulty	Quote
Being more person-centred	*GP: Well, it’s a bit paternalistic too. I mean, I explain how things work and how things are. But when I look back at it now, I do feel it’s a bit schoolmasterly. I: What could you have done differently? GP: Well, I could have done it more in the form of questions, because it’s debatable whether that’s how she perceives it. (GP 5)*
	*GP: Because she herself said, “Oh, these stomach pains, I want to do something about them.” At that point I could have asked, “What do you want to do? How do you feel about that?” Now I make a proposal, but of course she has to be OK with that. (GP 19)*
Structure of the consultation	*GP: Right, I realise I could have summarised things. And I could have been more explicit about the stages, saying OK, this is the moment to ask questions and then I’ll be doing the physical examination. I would have preferred it if I’d been clearer about that. (GP 10)*
	*GP: I’d have preferred to do that the other way round: first give the summary, then the conclusion, then the course of action. Now everything’s a bit mixed together so that makes it rather chaotic. I find that messy. (GP 16)*
A thorough psychosocial exploration	*I: What could you have done differently? GP: I could have spent a bit more time on the anxiety and emotions because now those aspects haven’t really been discussed fully. (GP 1)*
	*GP: I feel I didn’t ask her enough about why she’s so worried about the nausea. (GP 4)*

### Psychosocial exploration

Many GPs indicated that they should have paid more attention to exploring patients’ cognitions, ideas, concerns and thoughts regarding their symptoms in order to gain full insight into the patients’ experiences regarding their symptoms. They noticed that they had not done this as thoroughly as they should, because they thought this would save time. Some GPs said that they were reluctant to introduce a psychological explanation, which patients would resist.

*GP: Well, I actually think then, if I start summarising things at great length or asking even more questions, where does that get us in terms of time and the story? As it happens, I know I did consider that then, which is why I reckon I deliberately chose to do part of the medical history during the physical examination. Partly because I was under a bit of time pressure. How far did I go in asking her about the psychosocial aspects? Well, hardly at all really. We didn’t get onto that at all, whereas I think it is a key reason for her problem. Not that you can do much about it after the event, but we didn’t actually get onto that at all. (GP 16).*

### Structure of the consultation

Half of the doctors said that they should improve the structure of the consultation. According to GPs, a well-structured consultation would facilitate patients’ feeling of being taken seriously. As an example, one GP started with her explanation too early, before the physical examination. Other participating GPs experienced the consultation as chaotic: different stages of the consultation overlapped. As a consequence, GPs said that they were not sure whether they had a complete picture of the relevant topics. GPs said they should partition the different stages of the consultation more clearly by explicitly indicating when they were moving on to the next stage of the consultation. In order to improve the structure of the consultation further, GPs mentioned that they should make more use of summaries in which the patient’s question is reformulated.

*GP: I think it was basically a good consultation but the stages within it weren’t that clearly delimited; it was all a bit intermingled. I: Right, how could you have avoided that or what could you have done differently? GP: Well, by paying a bit more attention to it. So often when I want to move on to the next stage of the consultation, I should just summarise what we’ve discussed so far, and repeat the request for help. (GP 11).*

Some GPs mentioned they should discuss the consultation agenda more clearly. According to GPs, paying more attention to shared agenda-setting would help them to discuss what was considered to be really important for both the patient and the GP. However, some GPs struggled with agenda-setting as they were not sufficiently in control of the consultation.

*GP: Looking back, I realise that we each went into this consultation with our own agenda, and we should really have just discussed and agreed it jointly first. I think that’s what’s wrong. That could be an issue with other MUS patients too. Where the doctor and patient each have their own agendas. I think that’s something for me to look out for. I also think it’s important to be clear about that – just discuss it together, agree who’s going to go first and what we find important. I: Do you feel that you weren’t in control in this consultation? That you lacked a sense of where things were going? GP: Yes, yes. I: Why is that? GP: Because I simply don’t know where we’re heading and so items keep on coming up. I notice I’m not choosing between them and simply dealing with one item after another. So you get something that’s a bit inadequate. (GP 7).*

### Person-centredness

According to GPs, they should be more person-centred by giving attention to the reason for the appointment, making shared decisions, giving more room for the patient’s story and improving the quality of the contact. Some GPs mentioned that they should clarify the patient’s reason for the appointment more explicitly at the beginning of the consultation as they struggled with their consultation when the patients’ reason for coming along remained unclear. In such a situation, GPs said they tended to interrupt patients more frequently, left less room for the patient’s story and asked leading questions.

*GP: And I see myself struggling a bit. What should we talk about now? He does basically tell me things, and I notice that my questions can be rather leading and I can be a bit quick to cut in, where I could have given him rather more room. But, well, I think that’s because we basically always talk about the same things. (GP 3).*

Other GPs noticed they should have focused on the quality of their contact with the patient as this would have helped the patient to feel they were being heard. Some GPs noticed limited interaction with the patient, as they were “too busy with their computers”. They should have improved the quality of the contact by paying more attention to both verbal aspects, like giving feedback, and non-verbal communication elements, like eye contact, listening actively and nodding.

*I: What could you have done differently? GP: Well, like I just said, maintain contact with the patient rather than looking at the computer. I can imagine now that he might say the doctor showed a lack of interest in that last part. [...] I don’t see any genuine interaction in the entire last part. (GP 2).*

Some GPs mentioned that they should give more room to the patient’s story. Instead, they started to discuss their own ideas regarding the origin and treatment of the symptoms. For example, one GP said that she explained the symptoms in a paternalistic way without asking whether this applied to the patient. Another GP stated that he did not explain the link between the patient’s symptoms and the context as he thought the patient would not understand it. Other GPs said that they did not sufficiently involve the patient in the decision-making as they thought patients were not able to manage the symptoms by themselves. According to these GPs, they started to manage the patient’s problems according to their own judgment, while they now suggested it would have been better to involve the patient more actively.

*I: Because what happens now? GP: Well, I immediately go into ‘doctor mode’ and become doctor-centred. I should have given far more room for her own views on that large intestine and the constipation. I reckon that would have been better. I: What do you mean by doctor-centred? GP: That I become more proactive and start suggesting more things. I don’t give her much space to sort out her own problems because I don’t really have confidence that she’s capable of that. (GP 9).*

### Suboptimal communication as perceived by both the GP and the patient

GPs provided 84 comments on 60 fragments where they experienced difficulties. Analysis of the patients’ views on these fragments showed that in the majority of the fragments (*n* = 42) the patients’ comments were positive. Patients did not give any comment on nine fragments and they also experienced difficulties in the remaining nine fragments. The absence of a dialogue was a central feature in these fragments. This was characterised by the GP focussing on his/her own concept, asking closed questions and frequently interrupting the patient; the questions by the GP did not connect with what the patient was saying. Apparently, the GP followed some idea or concept in his/her own mind and had no intention of ‘following’ the patient. Examples are provided in Table 2, with the GPs’ and patients’ comments.

**Table 2:** Examples of the absence of a dialogue

GP: Right. I hear things aren’t going so well?	
P: No	
GP: No?	
P: Not at all. I phoned the GP out-of-hours surgery yesterday, actually. I got so short of breath I almost suffocated.	
GP: Right.	
P: So I don’t really trust the situation, to be honest.	
GP: Right, right, so where do you get that feeling that...?	
P: Here.	
GP: And do you feel feverish too? Do you feel sick a s well?	
P: My temperature went up but it’s gone back down. It’s just that I don’t have any energy.	
GP: OK, right.	
P: And sometimes I feel dizzy.	
GP: But I understand you’ve had these complaints for some time? Because if I look back a bit, you’ve had these complaints for several weeks.	
P: Yes.	
GP: Right.	
P: But not as bad as now.	
GP: So has it got much worse recently?	
P: Yes. Starting yesterday. And today it’s even worse.	
GP: Can you hear yourself wheezing too?	
P: No.	
GP: How do you find swallowing?	
P: Difficult.	
GP: Difficult. You don’t find yourself drooling?	
P: No, I don’t do that.	
GP: No. You’re still eating and drinking?	
P: Yes, though I find it difficult.	
GP: Are you still taking something for the pain?	
P: But I don’t feel any pain – it’s just suffocating.	
GP: Do you feel as though you’ve got a lump in your throat?	
P: Yes.	
**Quote GP:** “I think I’d have been better off asking about the purpose of the consultation because I can see that now I immediately start asking very specific questions about the complaints. Then I’m really focusing much too much on the complaints again rather than trying to find out what she wants help with.”	**Quote patient:** “Yes, I felt irritated because I had this feeling that the doctor wasn’t taking me seriously. I just didn’t feel I was being taken seriously. The GP should have discussed my question sooner and that rather gave the game away. The GP didn’t do that.”
GP: How are you physically, apart from that?	
P: Yeah, that’s fine.	
GP: But …?	
P: Except I was here a while back too for my stomach pain.	
GP: Right.	
P: And I had real problems yesterday and today with cramp. That was really painful cramp, you know.	
GP: Because you’ve been to hospital, right?	
P: Yes, but it was nothing. Fortunately they couldn’t find anything.	
GP: Nothing. So what is it, then?	
P: But that day I... I felt really terrible and I couldn’t even get up because of the pain. I was in an awful lot of pain then.	
GP: Right, exactly.	
P: But now it’s more like, I don’t know what it was, what do you call that? Because I used sachets and they didn’t help.	
GP: No.	
P: And I have this feeling that my stomach is clogged up or something because I’m also getting a lot of wind and nasty cramps.	
GP: Right.	
P: Yeah, incredibly painful cramps. And I’m also feeling sick, so it has been really bad today and yesterday.	
GP: Exactly. OK.	
P: So, there you have it.	
GP: Something new came on the market recently.	
P: Really?	
GP: Yes. It works on the basis of peppermint oil and it’s very much for irritable bowels like you have, so perhaps that’s something you could try. You haven’t taken it yet and it has only been around for a few months.	
**Quote GP** Of course, I take things much too quickly again with her, in the sense that I don’t explore things properly. That’s because I see her so often. She’s one of the patients who has been to the surgery most often since I’ve had the practice. I’ve seen her at least thirty times in the past 3 years. But yes, I take things too quickly here”.	**Quote patient:** “Then the doctor usually says it’s all in the mind; that’s happened a few times. Now I get the feeling he just takes a quick look. Yes, it’s you again … then quickly shows me the door. Then I don’t feel I’m being taken seriously”.

## Discussion

### Summary of main findings

In this study, GPs were asked to reflect on their own video-recorded MUS consultations. We identified 3 issues for improvement in the GPs’ communication: psychosocial exploration, structure of the consultation and person-centredness. The analysis of patients’ views on these fragments showed that in the majority of cases, the patients did not experience any problem. Those fragments in which patients also experienced problems were characterised by the absence of a dialogue.

### Comparison with literature

As far as we know, this is the first study where GPs were asked to reflect on their own MUS consultations in order to identify potential improvements in their communication. GPs said they should pay more attention to a thorough psychosocial exploration. Other research showed that GPs think that patients would resist a thorough psychosocial exploration [23]. This is allied to the incongruence between the GPs’ and the patients’ concept of disease in MUS, in which patients with MUS mainly have a biomedical model and GPs employ a psychosocial model [11, 23]. Furthermore, it is known that patients with MUS provide psychosocial cues and that most GPs disregard these cues [15]. We assume that a psychosocial exploration will not be resisted by patients only if GPs respond to their patients’ cues.

We found that GPs mentioned that they should have paid more attention to the structure of the consultation. This is in line with previous research, which showed that GPs experience difficulties in structuring their MUS consultations [24], that GPs tend to abandon the consultation structure and that they perceive symptom presentation as complex in patients with persistent MUS [25]. Further, paying more attention to the structure of the consultation could be related to the way doctors made questions at the same time they were reasoning. In previous research, Charlin et al. described the need to develop diagnosis scripts for certain vague symptoms [26]. We described in another research that non-analytical reasoning (i.e. clinical reasoning in which GPs do not explicitly test hypotheses) was a central component in their thought process in MUS consultations [21].

Further, GPs in our study indicated that they should communicate in a more person-centred way. Person-centredness is just like continuity of care one of the core values of primary care [27]. Other research showed that although GPs take more time for MUS consultations, they communicate in a less person-centred way with patients with MUS compared to patients with more medically straightforward presentations [16]. Giving attention to the reason for the appointment, making shared decisions, giving more room for the patient’s story and improving the quality of the contact are all closely related to the definition of person-centred care: care that takes into account the patient’s needs and preferences by exploring both the disease and illness experiences while understanding the whole person, finding common ground regarding management, and enhancing the doctor–patient relationship [28]. This is in line with a narrative review study, which used the available national guidelines on MUS and Cochrane Reviews [29]. All national guidelines recommend a thorough exploration, validation of the patient’s distress and providing insight into the patient’s biopsychosocial background, and stress the need for a shared understanding of the symptoms and the importance of the doctor-patient relation [29].

It is unclear whether GPs could improve communication by paying more attention to a thorough psychosocial exploration, improving the structure of the consultation and being more person-centred, because the majority of the patients did not experience any difficulties with these fragments of the consultations. The GPs were probably referring to an internalised ideal consultation model. By analysing the fragments in which both the patient and the GP experienced problems, we found that GPs might improve communication if they focused more on the dialogue and limited interrogation to a well-defined part of the consultation. A dialogue provides more opportunities to understand the patient’s illness experience [30]. This is in line with other research showing that in non-acute situations, patients prefer communication that gives voice to the life-world of the patient above communication characterised by a more technical interest [31]. Furthermore, the importance of giving space and encouraging the patient whatever he or she wants to tell the GP about the presented problem (inductive foraging, interpretive medicine) has been shown before [32]. The fragments where both GPs and MUS patients experienced suboptimal communication were almost equally distributed over the three identified themes in which GPs indicated they could improve the communication.

### Strengths and limitations

The strengths of this study are, first, minimisation of social desirability bias by using the method of stimulated recall. Secondly, we minimised recall bias by using the videos as a prompt to aid discussion. Thirdly, we used real-life consultation behaviour. Video-recorded consultations reflect habitual consultation behaviour because video-recording has no significant effects on the behaviour of patients or physicians [33, 34]. Although there are some limitations with stimulated recall method (memory access, creating a new ‘view’ by reflection, bias by the researcher) [35], we think that stimulated recall is the most appropriate method to achieve the goals of our study offering evidence that would otherwise be less valid and more difficult to obtain. Fourth, we used a qualitative analysis of the reflections of GPs with an iterative process of analysing and discussing until no new categories were found during the coding process. Finally, the data were analysed independently by three researchers. A possible limitation of this study is the selection of the participating GPs. Although we used purposive sampling to obtain participating GPs with different (clinical) backgrounds, it might be that GPs who refused to participate are less interested in MUS or have more negative attitudes and experience more or different difficulties during the consultations. As a consequence, this study might underestimate the difficulties of GPs in consultations with MUS patients. Further, the potential selection bias could partly be attributed to the video-recorded format. A second limitation is the selection of MUS patients. In contrast to many other studies, we identified patients as MUS based on the doctor’s opinion, rather than a validated questionnaire, for example. However, our purpose was to analyse GPs’ experiences when they have the diagnosis ‘MUS’ in their mind.

### Implications for clinical practice and further research

The results of our study emphasise the fact that effective physician-patient communication is of the utmost importance in the clinical encounter with MUS patients — including in the eyes of the GPs themselves. Still, there is room for improving GPs’ communication in MUS consultations. According to the participating GPs, they should pay more attention to a thorough psychosocial exploration, to the structure of the consultation and to being more person-centred. However, whether this leads to better care for MUS patients is open to question, as these findings did not match patients’ views. If GPs want to improve the communication in MUS consultations, they should probably also pay more attention to interaction in a dialogue and adopt less of an interrogative style in certain parts of the consultation.

Many GPs still experience MUS consultations as challenging. GPs tend to ignore psychosocial cues and do not provide a tangible explanation, resulting in unnecessary potentially harmful additional investigation. In addition, they make an incomplete diagnosis as they do not explore the patients’ ideas, expectations and cognitions in depth. As a consequence, they do not construct a meaningful narrative (a diagnosis in the biopsychosocial sense) about the patients’ symptoms. GPs must widen their focus through dialogue to understand the patients’ symptoms and to give a diagnosis in the biopsychosocial sense. This would imply that we have to train GPs better in how to manage patients with MUS. Previous research already showed that stimulated recall seems to be an important tool for caregivers in healthcare [36–38]. It gives them a better understanding of their actions which may help to improve their professional skills. This may be particularly valuable in managing MUS consultations as many GPs experience these consultations as challenging.

This study is part of a larger study that aims to develop an effective communication intervention GPs, as part of the regular consultation, that is acceptable for patients with MUS. A next step will be to develop the intervention and a training program in which we will teach GPs the intervention.

## Conclusion

GPs were aware of the importance of good communication. According to them, they could improve their communication further by paying more attention to psychosocial exploration, the structure of the consultation (more attention to summaries, shared agenda setting) and person-centredness (more attention to the reason for the appointment, the patient’s story, the quality of the contact and sharing decisions). The transcripts where the patient experienced problems too were characterised by the absence of dialogue.

## Additional file

**Additional file 1.** Interview guide: “What do you think of the consultation after watching it so far?” “What could you have done differently?”.

## Data Availability

Data are available at the Radboud University Medical Center, department of Primary and Community Care. The corresponding author can be contracted to access the data.

## Abbreviations

GP: General practitioner
MUS: Medically unexplained symptoms
