# Supplementary material for: Which difficulties do GPs experience in consultations with patients with unexplained symptoms: a qualitative study
Source: BMC Fam Pract. 2019 Dec 29;20:180. doi: 10.1186/s12875-019-1049-x (PMC6935475; doi:10.1186/s12875-019-1049-x)
Supplement: Supplementary file 1 — Additional file 1. Interview guide: “What do you think of the consultation after watching it so far?” “What could you have done differently?”. [file 12875_2019_1049_MOESM1_ESM.docx]

**Additional file 1**

Interview guide:

‘‘What do you think of the consultation after watching it so far?’’

’’What could you have done differently?’’
